# Assessing the validity of abbreviated literature searches for rapid reviews: protocol of a non-inferiority and meta-epidemiologic study

**DOI:** 10.1186/s13643-016-0380-8

**Published:** 2016-11-22

**Authors:** Barbara Nussbaumer-Streit, Irma Klerings, Gernot Wagner, Viktoria Titscher, Gerald Gartlehner

**Affiliations:** 1 Cochrane Austria, Danube University Krems, Dr.-Karl-Dorrek-Str. 30, 3500 Krems, Austria; 2 Department of Evidence-based Medicine and Clinical Epidemiology, Danube University Krems, Dr.-Karl-Dorrek-Str. 30, 3500 Krems, Austria; 3 RTI International, 3040 Cornwallis Road, Research Triangle Park, NC 27790 USA

**Keywords:** Rapid reviews, Abbreviated search, Streamlined search, Meta-epidemiologic study, Non-inferiority margin, Impact on conclusions

## Abstract

**Background:**

Systematic reviews offer the most reliable and valid support for health policy decision-making, patient information, and guideline development. However, they are labor intensive and frequently take longer than 1 year to complete. Consequently, they often do not meet the needs of those who need to make decisions quickly. Rapid reviews have therefore become a pragmatic alternative to systematic reviews. They are knowledge syntheses that abbreviate certain methodological aspects of systematic reviews to produce information more quickly. Methodological shortcuts often take place in literature identification. A potential drawback is less reliable results. To date, the impact of abbreviated searches on estimates of treatment effects and subsequent conclusions has not been analyzed systematically across multiple bodies of evidence. We aim to answer the research question: Do bodies of evidence that are based on abbreviated literature searches lead to different conclusions about benefits and harms of interventions compared with bodies of evidence that are based on comprehensive, systematic literature searches?

**Methods:**

We will use a non-inferiority and meta-epidemiologic design. The primary outcome is the proportion of discordant conclusions based on different search approaches. Drawing of a pool of Cochrane reports published between 2012 and 2016, we will randomly select 60 reports. Eligible reports are those that present a summary-of-findings table, draw a clear conclusion, present data for meta-analyses, and document the search strategy clearly. We will conduct several abbreviated searches to detect whether included studies in these Cochrane reviews could be detected. If searches could not detect all studies, we will revise the original summary-of-findings table and ask review authors whether the missed evidence would change conclusions of their report. We will determine the proportion of discordant conclusions for each abbreviated search approach. We will consider an abbreviated search as non-inferior if the lower limit of the 95% confidence interval of the proportion of discordant conclusions is below the non-inferiority margin, which is determined based on results of a survey for clinical and public health scenarios.

**Discussion:**

This will be the first study to assess whether the reduced sensitivity of abbreviated searches has an impact on conclusions across multiple bodies of evidence, not only on effect estimates.

## Background

Because of their high methodological standards in summarizing primary research, systematic reviews offer the most reliable and valid support for clinical and health policy decision-making, patient information, and guideline development. They aim to ensure methodological rigor. Systematic reviews are time- and labor-intense and thus are expensive. Because systematic reviews can take up to 24 months to complete [1, 2], they often do not meet the time-sensitive needs of decision-makers.

Consequently, rapid reviews have become a pragmatic alternative to systematic reviews. Rapid reviews are knowledge syntheses that simplify certain methodological aspects of systematic reviews. By doing so, they make results available in a shorter timeframe ranging from a few weeks to a few months [3]. The methodological underpinnings of rapid reviews are heterogeneous and employ diverse approaches with a strong focus on the specific needs of decision-makers [2, 4]. Although no universally accepted definition of rapid reviews exists, most methodological shortcuts when conducting a rapid review take place in those areas which are most time-consuming, in particular literature identification, quality assessment, and evidence synthesis [5]. A potential trade-off of rapid reviews is that their results can have a lower level of reliability than the results of systematic reviews. Studies comparing findings of rapid reviews with those of systematic reviews provide mixed results regarding concordance of conclusions [6–8].

A frequently employed approach in rapid reviews is to streamline literature searches [9]. Rapid review investigators often omit time-consuming search strategies such as searches in multiple electronic databases, handsearches, searches for gray literature, or citation tracking. Several methodological studies have shown that abbreviated search strategies do not detect the same amount of relevant studies that comprehensive systematic searches do [10–14]. The extra effort to find the totality of published studies, however, is often high. In a study on public health interventions for the prevention of cardiovascular disease, researchers found 31 of 34 studies with a search in MEDLINE only. Investigators had to search three additional electronic databases to detect the remaining three studies [13]. Likewise, in another recent study of streamlined searches using chronic depression as an example, investigators were able to identify 42 out of 50 studies by sensitive searches of electronic databases [15]. The remaining eight studies could only be found by supplementary search strategies such as citation tracking, screening reference lists, searching clinical trial registers, and contacting authors of included studies. The investigators concluded, however, that the studies missed by electronic database searches would not have had a significant impact on overall meta-analyses results and would not have affected conclusions. Furthermore, these studies were characterized by higher risks of bias. Likewise, Halladay et al. found that searches beyond PubMed for therapeutic interventions have little impact on meta-analyses results [14]. By contrast, a methods study with a focus on observational studies suggests that searches in single databases for observational studies do not provide comprehensive summaries of the existing literature [16].

The studies described above focused on single topics or changes in point estimates of meta-analyses. To date, the question whether the reduced sensitivity of abbreviated searches has an impact on estimates of treatment effects (beneficial and harmful) and subsequent conclusions has not been analyzed systematically across multiple bodies of evidence.

## Methods

### Aim and research question

The aim of the study is to assess the impact of various streamlined searches on conclusions about benefits and harms of an intervention. We will focus on two areas of interest: (1) clinical topics for which randomized controlled trials tend to provide the most reliable evidence and (2) public health topics for which randomized controlled trials are often not available and evidence comes from observational studies.

With our study we want to answer the following research question.

Do bodies of evidence that are based on abbreviated literature searches lead to different conclusions about benefits and harms of interventions compared with bodies of evidence that are based on comprehensive, systematic literature searches?

### Study design

The study will use a non-inferiority and meta-epidemiologic design to test whether different abbreviated searches are non-inferior to (i.e., not worse than) comprehensive, systematic literature searches. The primary outcome of interest is the proportion of discordant conclusions based on different search approaches for the same key questions. Conclusions based on comprehensive systematic literature searches will serve as the reference standard. We denote the conclusions based on comprehensive systematic literature searches as *C*_S_ and those based on various abbreviated searches as *C*_A(1−*k*)_. The proportion of discordant conclusions based on the two search approaches would then be:

$$ \theta = \left({C}_{\mathrm{S}\ \hbox{-} }{C}_{\mathrm{A}\left(1\hbox{-} k\right)}\right)/n, $$

where *n* is the number of conclusions.

The non-inferiority margin (*ε*) in our study reflects the maximum risk of getting an incorrect answer from a rapid review that decision-makers are willing to accept in exchange for getting the product rapidly. We define an incorrect answer as a result that is different enough in direction or magnitude of effect compared with a result from a systematic review that the difference would trigger a signal to update a systematic review based on a modified approach by Shojania et al. [17] (Table 1).

**Table 1:** Criteria for an “incorrect answer” based on difference in statistical significance and magnitude of effect

Criteria for incorrect answer	
Change in statistical significance	Statistical significance changes between graded effect and gold standard effect (trivial changes in *p* values within the range of 0.04 to 0.06 are not considered as a change).
Change in magnitude of effect	Difference in magnitude of effects is larger than a relative risk change (increase or reduction) of 25 percentage points for dichotomous outcomes or 0.20 SMDs for continuous outcomes.

Incorrect answers of rapid reviews might lead to false conclusions. For the purpose of our study, we define false conclusion as a conclusion that is different from a conclusion based on a body of evidence derived from a comprehensive systematic literature search.

The null hypothesis of our study is denoted as *θ* > *ε* (abbreviated searches are inferior), whereas the alternative hypothesis can be denoted as *θ* ≤ *ε* (abbreviated searches are non-inferior).

**Fig. 1:**
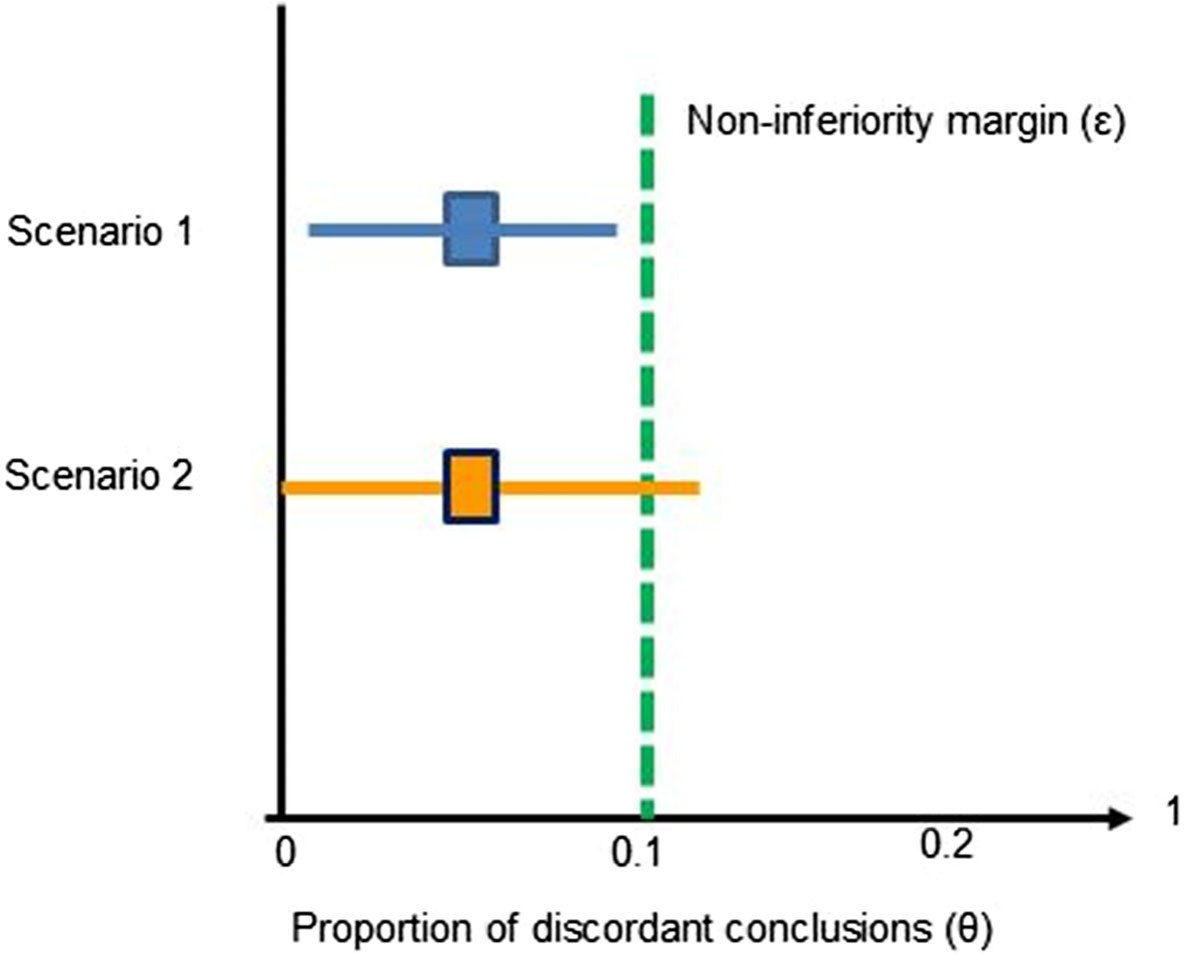
Two different possible results of a non-inferiority study comparing abbreviated searches with systematic searches

We will consider an abbreviated search as non-inferior if the lower limit of the 95% confidence interval of the proportion of discordant conclusions is below the non-inferiority margin of 0.1. With a non-inferiority margin of 0.1 (i.e., decision-makers are willing to accept an incorrect answer in one out of ten decisions based on a rapid review), we would conclude that abbreviated searches are non-inferior if the lower confidence interval of discordant conclusions does not cross this threshold. Figure 1 depicts two possible scenarios. In scenario 1, the lower limit of the confidence interval does not cross the non-inferiority margin, demonstrating non-inferiority of abbreviated searches relative to the gold standard of systematic searches. In scenario 2, the lower confidence limit crosses the non-inferiority margin. We would conclude that abbreviated searches are inferior.

### Determination of non-inferiority margin

We recently conducted an international, online survey with stakeholders and decision-makers in the health care sector. The aim of the study was to determine the maximum risk of getting an incorrect (wrong or inaccurate) answer from a rapid review that decision-makers are willing to accept in exchange for a rapid evidence synthesis. The survey targeted guideline developers and decision-makers who work in regulatory agencies, health insurance companies, or health policy agencies and have experience using evidence summaries. Survey language was English, German, and Spanish. The survey has now closed and 334 persons from 33 countries have participated. Preliminary results show that, on average, respondents are willing to accept a maximum risk of getting an incorrect answer of 10% (median).

We determined the non-inferiority thresholds based on results of this survey for clinical and public health scenarios.

### Determining sample size

Based on the non-inferiority margin, we conducted sample-size calculations for the number of Cochrane reviews needed using Simon’s two-stage design [18]. Table 2 presents required sample sizes of Cochrane reviews for different non-inferiority margins for correlated marginal proportions. All calculations are based on a significance level of 0.025 and a power of 0.9. Based on the 10% non-inferiority margin, we require a minimum of 60 Cochrane reviews.

**Table 2:** Exemplary sample size calculations for different non-inferiority margins

Non-inferiority margin	Required sample size
2%	516
3%	313
5%	139
7%	86
10%	60
12%	50
15%	30

### Eligible evidence base

We will choose Cochrane reports that fulfill the following inclusion criteria:

1. Authors were able to draw a conclusion about the effectiveness or risk of harms of a treatment compared with a control intervention. We will exclude Cochrane reports that state that the evidence is insufficient to determine efficacy, effectiveness, or risk of harms.
2. Relevant outcomes are presented in a summary-of-findings table including grades of the quality of evidence for each outcome according to the GRADE (Grading of Recommendations Assessment, Development and Evaluation) approach.
3. Data for meta-analyses are clearly presented to be able to reproduce the analyses. We will also include Cochrane reports that couldn’t calculate a meta-analysis, because only one study was identified, if the authors were able to draw a conclusion based on this study.
4. Searches are reported in enough detail to replicate the search strategy in selected databases.
5. The most recent literature search was run in 2012 or later, and the date of the searches (at least months and year) is reported.
6. The review focuses on one of the following clinical topics or on a public health topic:
  Cardiovascular disease (e.g., myocardial infarction)
  Cerebrovascular disease (e.g., stroke)
  Osteoarthritis
  Chronic respiratory conditions (e.g., chronic obstructive pulmonary disease)
  Mental health

We narrowed the scope of clinical topics to disease categories that appear on numerous authoritative lists of “priority conditions” from e.g., the Institute of Medicine, the Centers for Disease Control and Prevention, or the Agency for Healthcare Research and Quality. We will not narrow the scope of public health topics, because only a limited number of public health Cochrane reviews have been published.

### Identifying the evidence base

We will systematically search the Cochrane Library from 2012 to 2016 to find Cochrane reports that present the quality of evidence in summary-of-findings tables. We chose 2012 as start date because with using CRS Online interface it is only possible to identify when a record was added to CENTRAL for records added after 2011. According to the Methodological Expectations of Cochrane Intervention Reviews (MECIR), summary-of-findings tables are highly desirable but not mandatory [19]. Consequently, not all Cochrane reviews present them. We will use “quality of evidence” OR “summary of findings” as search terms. Out of the pool of unsorted Cochrane reports with summary-of-findings tables, we will assign a random number to each Cochrane review that was identified by our searches. We will then rank the reviews by random number and apply our eligibility criteria sequentially to each review until we have at least 60 reviews. Ranking the reviews by random numbers helps us to avoid bias due to publication date, because it makes the order of reports that we screen for eligibility random. For the identification of eligible Cochrane reports, we will use a pilot-tested review form.

Because the change in conclusions is the primary outcome of interest, we will focus on all outcomes (dichotomous and continuous) presented in the main summary-of-findings table. Outcomes presented in the summary-of-findings tables are usually those that reviewers viewed as most important for decision-making.

### Data collection

For each eligible review, we will extract bibliographic information about included studies. We will extract general information about the publication (authors, journal, publication year, etc.) and database-specific identification numbers. We will then check whether the included publications (if more than one publication is available for a given study, we will search for each publication that is included in the Cochrane report) could have been detected at the date of the search of the respective Cochrane report with only the following sources searched:

- PubMed or MEDLINE only
- CENTRAL (Cochrane Central Register of Controlled Trials) only
- Embase only
- PubMed or MEDLINE plus CENTRAL
- PubMed or MEDLINE plus Embase
- CENTRAL plus Embase
- PubMed or MEDLINE plus CENTRAL plus Embase
- PubMed or MEDLINE plus reference lists
- CENTRAL plus reference lists
- Embase plus reference lists
- PubMed or MEDLINE plus CENTRAL plus reference lists
- CENTRAL plus Embase plus reference lists
- PubMed or MEDLINE plus CENTRAL plus Embase plus reference lists
- PubMed or MEDLINE plus Embase plus reference lists

The database choice is based on the Methodological Expectations of Cochrane Intervention Reviews (MECIR) which demand that MEDLINE, Embase, and CENTRAL are searched [19]. We chose these combinations of databases and reference tracking because a study exploring methods of rapid reviews reported that the majority of studies looking at abbreviated searches only searched one database with or without additional handsearches of reference lists of included studies [4]. Thus, it is unknown whether the abbreviated searches could affect conclusions of reviews.

To identify publications in the respective databases, we will either use database-specific identification numbers (PubMed ID (PMID), Central ID, Embase Accession Number) if available, or search strings combining author names, title words, and additional bibliographic information (journal of publication, year, volume, etc.). We will also limit the search results by entry date to determine whether the record was available at the time of the original search (as defined by the date of the last search documented in the review).

In a second step, we will rerun the original MEDLINE, CENTRAL, and Embase searches documented in the review. We will then test if the references present in the databases were identified by the search strategy. This step is important because recall, defined as the number of studies that are actually identified through search strategies, is usually below the total possible coverage. One study suggests that e.g., in Medline only about three quarters of listed studies could actually be identified by search strategies [20]. Or, if available, we will use the archived search results provided by Cochrane information specialists, who conducted the search for the Cochrane review. We will do formal quality assessment of search strategies: spelling, syntax and operators of searches will be checked and minor errors will be corrected.

Reference lists of included studies found in each database will be exported from Scopus. To identify Scopus records, we will use PMID if available, or search strings combining author names, title words and additional bibliographic information (journal of publication, year, volume, etc.).

We will determine the proportion of studies included in the Cochrane reviews that were detected with each abbreviated search approach.

### Data analysis

To determine whether approaches are non-inferior to comprehensive systematic searches, we will employ qualitative and quantitative methods. We will assess concordance of conclusions as well as concordance of effect estimates. We will contact the Cochrane Central Editorial team to provide us with the original RevMan files for each selected Cochrane report. RevMan is the software Cochrane authors use to write and edit their reviews.

#### Assessing concordance of conclusions

We will compare effect estimates in Cochrane reviews with those from studies identified in abbreviated literature searches. We will conduct meta-analyses with the eligible studies detected by each abbreviated search approach using the same statistical approach as the respective Cochrane report. For each abbreviated search, team members experienced with GRADE will revise the summary-of-findings table from the underlying Cochrane report and update number of studies, and effect estimates, but not the grading of the quality of evidence. We will highlight differences between original summary of findings tables and those based on abbreviated searches in comparison tables.

We will contact review authors of the underlying Cochrane reviews and ask them for a judgment whether the missed evidence would change conclusions of their report. We would like them to decide whether the evidence base from abbreviated searches changes their conclusion, to keep it consistent and avoid bias due to different judge. We will supply them with the revised summary of findings tables and the comparison tables.

Review authors will have the choice between two answers:

1. The body of evidence based on an abbreviated search would lead to the same conclusion (concordant conclusion).
2. The body of evidence based on an abbreviated search would lead to a different conclusion (discordant conclusion).
  If authors opt to change their conclusions, we will ask them to indicate whether:
  - They have made the conclusion less definitive, but maintained the direction (positive or negative) of the original conclusion (e.g., by adding adverbs of probability such as “maybe,” “might,” etc.).
  - They could no longer draw a conclusion.
  - They have changed the direction of the conclusion (made a positive conclusion negative or a negative one positive) but also included an adverb to make the conclusion less definitive (e.g., from “is effective or might be effective” to “might not be effective”).
  - They have changed the direction of the conclusion (made a positive conclusion negative or a negative one positive), and state the newly derived conclusion in absolute terms (e.g., from “is effective or might be effective” to “is not effective”).

We will pilot our approach to ensure high response rates and valid responses.

As outlined above (see Fig. 1), we will then determine the proportion of discordant conclusions for each abbreviated search approach for clinical and public health conditions and assess whether the lower limit of the confidence interval crosses the non-inferiority margin.

#### Assessing concordance of effect estimates

To assess concordance of effect estimates, we will compare effect estimates in Cochrane reviews with those from studies identified in abbreviated literature searches. For this analysis, we will focus on one primary outcome for efficacy and harm of each included Cochrane report. In case, there are more eligible outcomes in one report, we will use the primary outcome based on most studies. We will only include dichotomous outcomes. We do not include continuous outcomes for this analysis because thresholds for clinical relevance are often difficult to determine. They would, for example, require information on minimal important differences, which is not available for many scales. To obtain the same direction of effects across all meta-analyses, we will recalculate effect estimates, if necessary, so that all results will be expressed as odds ratios of desirable results (e.g., response to treatment instead of failure to respond, survival instead of mortality). An odds ratio of less than 1.0 will represent undesirable effects, an odds ratio of larger than 1.0 will represent desirable treatment effects.

We will compare differences in effect estimates between the Cochrane reports and the evidence detected by streamlined search approaches using ratios of odds ratios and random effects models. To achieve an overall estimate of the difference, we will pool the ratios of odds ratios. Overall, we will follow a method outlined by Sterne et al about meta-epidemiological research [21]. In addition, we will determine the percentage weight contributed by studies that abbreviated searches did not detect to individual meta-analyses in the Cochrane reports.

## Discussion

To the best of our knowledge, this will be the first study to assess whether the reduced sensitivity of abbreviated searches has an impact on conclusions across multiple bodies of evidence, not only on effect estimates. The strengths of our study are

- that we involve review authors and ask them to reassess their original conclusions based on a new evidence base generated by abbreviated searches,
- that we quantify the non-inferiority of abbreviated searches by employing a non-inferiority margin assessed by a survey of health policy decision-makers and guideline developers.

The project does not aim to abolish full systematic reviews but to provide a rationale for or against the use of rapid reviews where these are the only seemingly feasible way to support decision-making.

## Abbreviations

*C*_A(1-*k*)_: Conclusion based on various abbreviated searches
CENTRAL: Cochrane Central Register of Controlled Trials
*C*_S_: Conclusions based on comprehensive systematic literature
GRADE: Grading of Recommendations Assessment, Development and Evaluation
ID: Identification
MECIR: Methodological Expectations of Cochrane Intervention Reviews
*n*: Number of conclusions
PMID: PubMed identification
*ε*: Non-inferiority margin
*θ*: Proportion of discordant conclusions based on the two search approaches
